# Fourier Ptychographic Microscopy Reconstruction Method Based on Residual Local Mixture Network

**DOI:** 10.3390/s24134099

**Published:** 2024-06-24

**Authors:** Yan Wang, Yongshan Wang, Jie Li, Xiaoli Wang

**Affiliations:** Electronics Information Engineering College, Changchun University, Changchun 130022, China; wangy8512@ccu.edu.cn (Y.W.); lij69@ccu.edu.cn (J.L.); wangxl@ccu.edu.cn (X.W.)

**Keywords:** FPM, noise, channel attention, spatial attention, diffusion model

## Abstract

Fourier Ptychographic Microscopy (FPM) is a microscopy imaging technique based on optical principles. It employs Fourier optics to separate and combine different optical information from a sample. However, noise introduced during the imaging process often results in poor resolution of the reconstructed image. This article has designed an approach based on a residual local mixture network to improve the quality of Fourier ptychographic reconstruction images. By incorporating channel attention and spatial attention into the FPM reconstruction process, the network enhances the efficiency of the network reconstruction and reduces the reconstruction time. Additionally, the introduction of the Gaussian diffusion model further reduces coherent artifacts and improves image reconstruction quality. Comparative experimental results indicate that this network achieves better reconstruction quality, and outperforming existing methods in both subjective observation and objective quantitative evaluation.

## 1. Introduction

Spurred by the unceasing development of scientific and technological capabilities, the process of delving into the microscopic domain has become ever more in-depth. Researchers in biology, medicine, materials science, and other fields require more precise observation and measurement of microscopic structures to better understand their characteristics, functions, and interactions. Traditional microscopy techniques are often limited by the diffraction limit of light and sample scattering, which prevents them from providing high-resolution images. In 2013, Guoan Zheng et al. proposed a new quantitative imaging technique called Fourier Ptychographic Microscopy (FPM) [1,2]. Unlike traditional microscopes, this technique illuminates the sample with different incident angles sequentially and captures a set of low-resolution images with corresponding spatial spectra. The low-resolution intensity images obtained from different angles are then iteratively processed in the Fourier domain to solve for the optimal solution that satisfies both the spatial amplitude constraint and the frequency domain support constraint, thereby reconstructing the high-resolution image of the sample [3,4].

Despite its numerous advantages, FPM faces some challenges and limitations. The imaging speed is relatively slow, necessitating improved scanning and computational methods to enhance efficiency. The reconstruction process can also be affected by noise and system errors, requiring more effective denoising and optimization algorithms to improve image quality. Traditional FPM algorithms are mainly based on frequency domain processing and back-projection principles. They process the frequency domain data of the sample and then use back-projection algorithms to reconstruct the three-dimensional structure of the sample. For instance, Zuo et al. [5] and Bian et al. [6,7] manually adjusted parameters and performed multiple iterations to improve noise robustness, accelerate the convergence of the reconstruction process, and enhance reconstruction results. However, traditional reconstruction algorithms take a long time due to multiple iterations. The neural network-based approach employs a large-scale pre-established dataset to train an end-to-end deep convolutional neural network, which is used to reconstruct high-resolution intensity and phase images. Jiang et al. [8], Sun et al. [9], and Zhang et al. [10] solved for high-resolution intensity and phase images using the backpropagation of neural networks. However, neural network modeling methods [11,12,13] are essentially gradient descent-based iterative methods and do not completely overcome the drawbacks of iterative algorithms, such as slow reconstruction speed and susceptibility to noise.

Although traditional neural network algorithms can reconstruct high-resolution images, they still have some shortcomings. During the course of recent years, image processing approaches powered by deep learning have advanced rapidly, gradually extending from fields like object recognition and image classification to image super-resolution and other image reconstruction areas [14,15,16,17,18]. Dong et al. [19] first applied deep neural networks to image super-resolution, surpassing a series of traditional algorithms, like sparse coding [20] and neighbor embedding [21], in image reconstruction quality. In Kim et al. [22], the concept of residual learning was first introduced [23,24,25], reconstructing the missing high-frequency residuals in low-resolution images to improve image resolution. Sun et al. [26] put forth the double-flow convolutional neural network (DFNN) approach, which supplanted traditional iterative approaches to improve the quality of single wide-field reconstructed images, but this method could not be used for large-scale image reconstruction, limiting its scope. Sun et al. [27] put forth the neural network model combined with pupil recovery (FINN-P) approach, which uses a more efficient workflow and a selection of dissimilar optimizers in the imaging network, achieving better reconstruction results than the neural network-based method by Jiang [8]. Moreover, deploying the proposed network on neural engines or tensor processing units (TPUs) has the potential to further enhance the reconstruction speed of FINN-P. Zhang et al. [28] put forth the integration of neural network and physical reconstruction model (FuNN) method, fusing FPM’s physical reconstruction model with a convolutional neural network and optimizing the weights and biases of FuNN. Compared to FINN-P, FuNN does not require alternating training processes with different network settings, thus speeding up the reconstruction process. Zhang et al. [29] proposed the physics-based learning with channel attention (PbNN-CA) method, integrating a physics-based network with a channel attention module (CA). Integrating the CA module into the physics-based network renders it capable of correcting pupil aberrations and LED intensity errors at the same time. Employing the channel attention module enhances the performance and noise resilience of PbNN. Our team published a Fourier ptychographic reconstruction method based on a residual hybrid attention network in 2023 [30], combined channel attention with spatial attention, apportioning channel weights and procuring high-resolution spatial features founded on residual learning to ameliorate the quality of image reconstruction.

The article describes a Fourier ptychographic reconstruction solution, which was developed based on a residual local mixture network. By introducing a mixture attention mechanism and combining it with a Gaussian diffusion model, the quality of the reconstructed images generated by this network is vastly enhanced. The mixture attention mechanism allows the model to utilize both channel and spatial information simultaneously, while the Gaussian diffusion model simulates the diffusion process of images in the Fourier space, effectively handling noise and artifacts in microscopic images. By integrating the mixture attention mechanism with the Gaussian diffusion model, we can suppress noise interference while preserving image details, resulting in clearer microscopic reconstruction images. Comparative experimental analysis verifies that our approach attains considerable advancements in the quality and the efficiency of the reconstructed images. Our foremost contributions can be set forth as follows:

1. To address the issues of poor reconstruction quality and low efficiency, a mixture attention network is introduced to optimize the reconstruction efficiency, reduce computational complexity, improve quality, and minimize time costs;
2. To tackle the problems of noise and artifacts during the reconstruction process, a Gaussian diffusion model is introduced to simulate data diffusion, smoothing out noise, reducing coherent artifacts, and enhancing the quality and accuracy of reconstructed images.

## 2. Methods

### 2.1. Network Architecture

**Figure 1:**
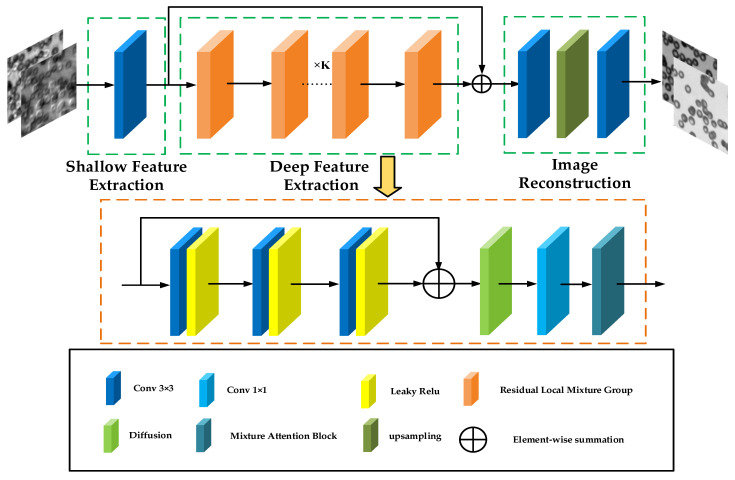
Structure of residual local mixture network.

The residual local mixture network (RLMN) is primarily composed of three parts, as demonstrated in Figure 1. The shallow feature extraction module employs a 3 × 3 convolution to extract shallow features through convolution operations, maintaining texture and local detail information while diminishing the likelihood of overfitting. The information obtained from the shallow feature extraction module is subsequently fed into the deep feature extraction as the input. In the deep feature extraction stage, K local residual fusion groups are embedded to effectively extract and process high-frequency information in the images. Additionally, to enrich the semantic information in the images, high-frequency information is combined with low-frequency information through residual connections. This enhances the richness of semantic information contained in the image. Concluding the process, the reconstruction module deploys upsampling module and two 3 × 3 convolutions to execute the objective of reconstructing high-frequency information. Included among these, the input data consist of a series of low-resolution images. These low-resolution data are then synthesized in the Fourier domain, and transformed into a dual-channel data format through inverse Fourier transform. This dual-channel data are then used as the input to the network.

### 2.2. Residual Local Mixture Group

As demonstrated in Figure 1, inside the deep feature extraction block, the input is first directed into a convolution module comprising a 3 × 3 convolution and a LeakyReLU activation function. The convolution module can enhance the model’s feature representation capabilities through the mechanisms of convolution and activation functions. Afterwards, the model inputs the information into the Gaussian diffusion model, simulating the image diffusion process in the Fourier space to handle noise and artifacts in the image. Following a 1 × 1 convolution to reduce channel dimensions, the information is then fed into the hybrid attention module to further enhance the quality of the reconstructed images.

### 2.3. Mixture Attention Block

**Figure 2:**
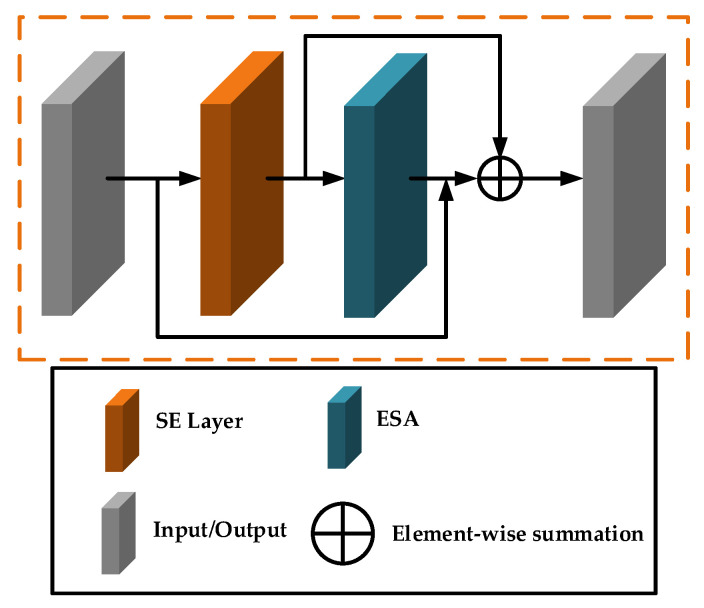
Structure of mixture attention block.

The spatial attention is enhanced by introducing channel attention into the spatial attention. First, the input is passed through the channel attention module, which distinctly characterizes the dependencies among channels to generate weights for each individual channel. These extracted weights are then applied to the feature maps for further processing. Subsequently, spatial attention focuses on the dependencies between different spatial positions in the feature maps to extract fine spatial features. The structure of the mixture attention block is portrayed in Figure 2.

### 2.4. Enhanced Spatial Attention (ESA)

**Figure 3:**
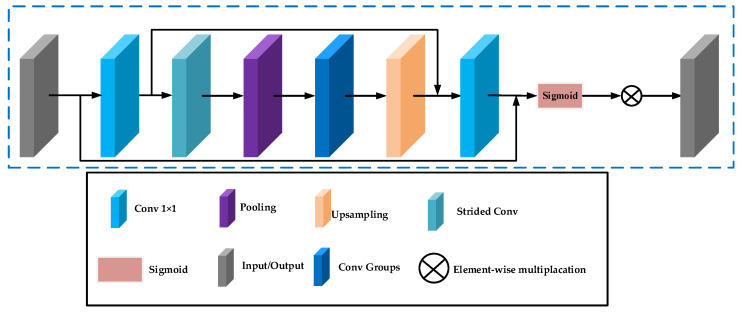
Structure of enhanced spatial attention.

As demonstrated in Figure 3, the spatial attention technique first leverages a 1 × 1 convolutional layer to lower the channel dimension. Then, to expand the receptive field, a combination of stridden convolution (stride 2) and max pooling layers are leveraged to swiftly downscale the spatial dimensions of the network. To restore the spatial size, an upsampling layer is added, and a 1 × 1 convolutional layer is leveraged to reinstate the channel count. Finally, a sigmoid layer is used to generate the attention mask. Furthermore, skip connections are introduced, enabling the direct transfer of high-resolution features preceding the spatial dimension reduction to the output of the block. This design not only optimizes the efficiency and effectiveness of the network but also achieves effective processing of information at different scales through fine-grained structural adjustments, thereby improving the overall performance.

### 2.5. SE Layer

**Figure 4:**
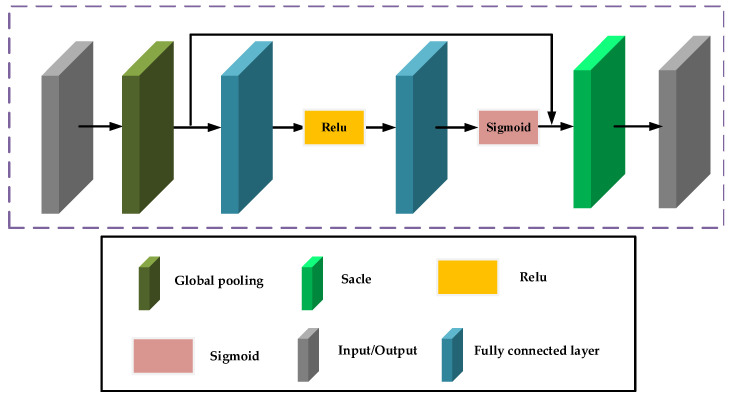
Structure of SE layer.

As demonstrated in Figure 4, the structure of the SE layer evaluates the importance of the distinct channels comprising the feature map and adjusts the channel weights based on these evaluations. The SE layer consists of two key operations: squeeze and excitation. First, the input information is passed through global average pooling to transform the feature map of each channel into a single real-valued quantity, reflecting the comprehensive information of that channel. This process captures the global receptive field of each channel, enabling the learning of dependencies between channels. Subsequently, a fully connected network (that comprises two dense layers and an activation function, with the first dense layer reducing the dimensions and the second restoring the original channel count) processes the real number sequence obtained from the squeeze operation. The fully connected network learns the importance of each channel and outputs a weight vector corresponding to the number of input channels. Finally, the SE layer multiplies the weight vector obtained from the excitation operation with the original feature map on a per-channel basis, thus adjusting the weights of the distinct channel features. The SE layer helps the network focus better on important feature channels and suppress less important ones, thereby improving the network’s performance.

### 2.6. Diffusion Model

**Figure 5:**
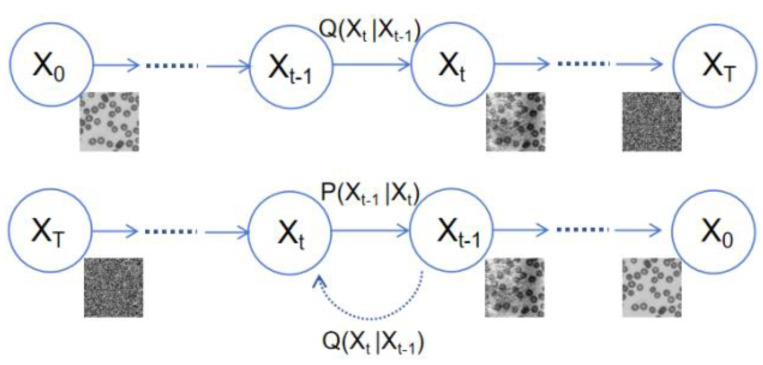
Diffusion model, with the noising process above and the denoising process below.

The diffusion model evolves from an initial state to a target distribution through a series of iterative processes. It can be seen as a random walk in probability space to simulate the target distribution. The diffusion model is significant in image-generation tasks. Leveraging its ability to learn the probability distribution of images can produce images that are of high quality and exhibit a high degree of realism. The diffusion model comprises two processes: “noising” and “denoising.” As shown in Figure 5, during the noising process, input X_0_ constantly mixes with Gaussian noise. After T iterations of noising, the image X_T_ becomes a pure noise image following a standard normal distribution. During the denoising process, the network learns T denoising steps to restore X_T_ to X_0_.

### 2.7. Evaluation Metrics

As a method of image reconstruction, Fourier ptychographic imaging directly reflects the effectiveness of the reconstruction algorithm as reflected in its reconstruction outcomes. The quality of image reconstruction is evaluated in this paper using the Peak Signal-to-Noise Ratio (*PSNR*) and Structural Similarity Index (*SSIM*) as the assessment metrics.

The Peak Signal-to-Noise Ratio (*PSNR*) in signal processing denotes the proportion between the maximum achievable signal power and the noise power that impacts the fidelity of the signal representation. In image evaluation, it can be defined by the Mean Squared Error *(MSE*):

(1) $$PSNR=-10\cdot \log\left(MSE\right)$$

The mean square error can be expressed as

(2) $$MSE=mean\left({\left(I_{1}-I_{2}\right)}^{2}\right)$$

where the variable $I_{1}$ equates to the authentic image, and the variable $I_{2}$ equates to the reconstructed image; a *PSNR* value that is more elevated corresponds to better image quality.

The Structural Similarity Index (*SSIM*) is given by:

(3) $$SSIM=\frac{\left(2\mu_{1}\mu_{2}+C_{1}\right)\left(2\sigma_{12}+C_{2}\right)}{\left({\mu_{1}}^{2}+{\mu_{2}}^{2}+C_{1}\right)\left({\sigma_{1}}^{2}+{\sigma_{2}}^{2}+C_{2}\right)}$$

where $\mu_{1},\;\sigma_{1},\;$ and $\mu_{2},\;\sigma_{2}$ are the mean and standard deviation of 1 and 2, respectively;${\;\sigma }_{12}$ is the covariance of the two; and $C_{1}=C_{2}\;$is a constant.

## 3. Experiment

### 3.1. Dataset

**Figure 6:**
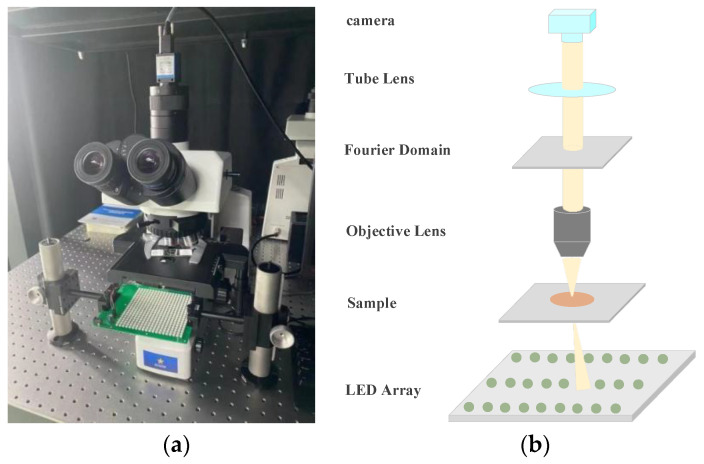
Fourier ptychographic microscope: (**a**) real FPM system; (**b**) FPM simulation schematic.

This experiment employs a simulated dataset encompassing 15,000 sets of high-resolution image data. Every set is composed of two images, one representing the intensity channel and the other the phase channel. The synthesized complex amplitude data from the two input channels are simulated using the Fourier imaging system. As part of the simulation process, Gaussian noise with a mean of 0 and a standard deviation of 3 × 10^−4^ is incorporated to emulate potential system error noise encountered in real-world imaging scenarios. These Gaussian noise-added data are regarded as low-resolution data pertinent to Fourier ptychographic imaging. A conventional Fourier ptychographic reconstruction algorithm, executed with a solitary iteration, is subsequently applied to combine these low-resolution data into low-resolution complex amplitudes. This produced 15,000 sets of low-resolution input data. Ultimately, the 15,000 sets of high-resolution image data were reserved to be used as the reference images for subsequent performance evaluation and comparative analysis. The architectural design of the Fourier ptychographic microscopy system is depicted in Figure 6.

### 3.2. Optimizer Comparison Experiment

**Figure 7:**
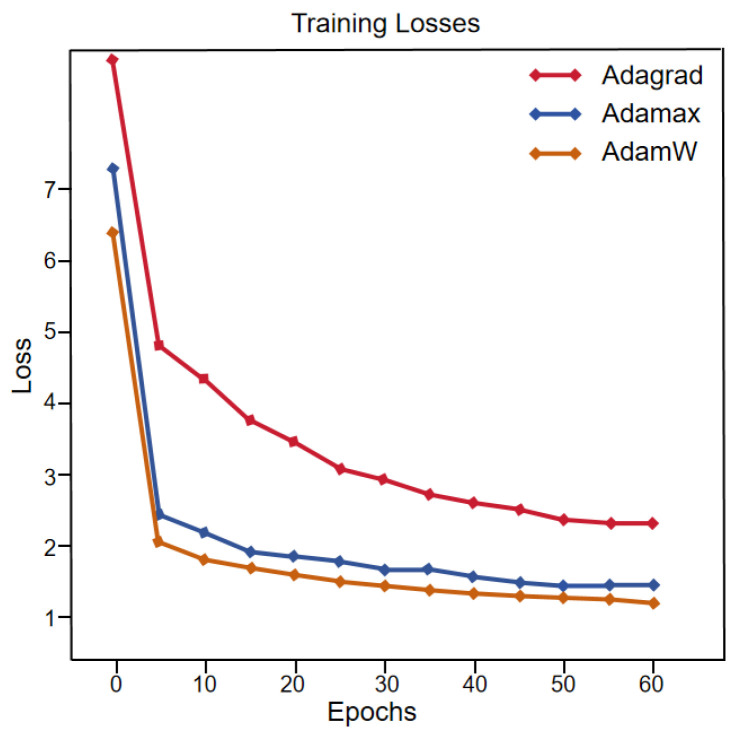
Loss curve comparison for different optimizers.

**Figure 8:**
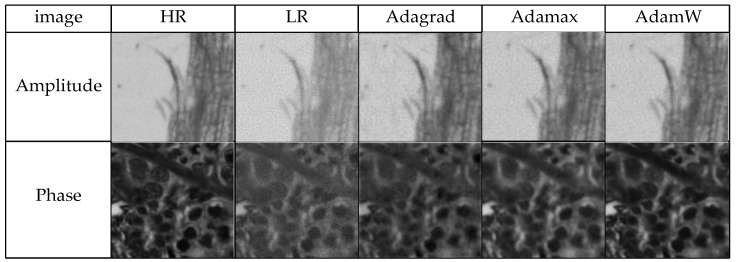
Reconstruction results for different optimizers.

To achieve better optimization results for the experiment while keeping the loss function and number of iterations the same, Adagrad, Adamax, and AdamW optimizers were chosen for comparison, respectively. The comparison of loss curves for different optimizers is shown in Figure 7. The number of iterations is shown on the horizontal axis, and the loss value for each training session is plotted on the vertical axis of this figure. The three curves represent the three different optimizers. From the figure, it is evident that the model trained using the AdamW optimizer demonstrates quicker loss decrease and superior convergence relative to the other two optimizers. The reconstruction findings are presented in Figure 8, where HR represents the original high-resolution image, and LR corresponds to its corresponding low-resolution image. It is discernible that the intensity images reconstructed by all three optimizers achieve good results, but the network trained with the AdamW optimizer produces the highest-quality phase images, followed by Adamax, with Adagrad yielding the lowest quality. Tabulated in Table 1 are the quantitative metrics, SSIM, and PSNR, for the reconstruction images generated by the three optimizers. As can be observed from the table, the AdamW optimizer significantly outperforms the other two optimizers in image quality. For intensity images, the PSNR of the AdamW reconstruction results is higher than the other two optimizers by 0.96 and 6.91, respectively. For phase images, the SSIM of the AdamW reconstruction results is higher than the other two optimizers by 0.0676 and 0.43, respectively. This comparative experiment serves to substantiate the effectiveness of the AdamW optimizer network.

### 3.3. Ablation Experiment

**Figure 9:**
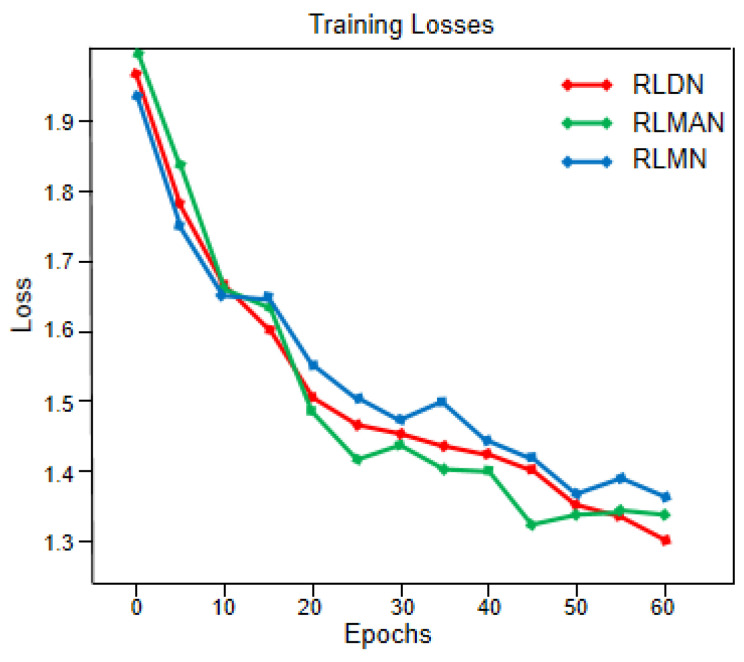
Loss curve comparison for different models.

**Figure 10:**
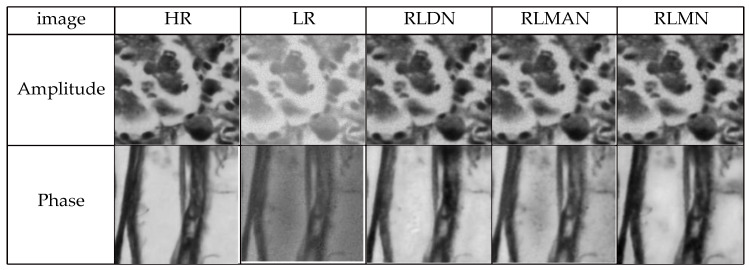
Reconstruction results for different models.

In this section, we conducted ablation experiments to validate the effectiveness of the Fourier ptychographic reconstruction method combining hybrid attention and Gaussian diffusion models. Among them, RLDN is the network with the standalone addition of the Gaussian diffusion model, RLMAN is the network with the standalone addition of the hybrid attention mechanism, RLMN is the residual local mixture network. Keeping the loss, learning rate, and noise parameters uniform, the network is trained using the three models. As presented in Figure 9, three model networks achieve good convergence performance, but the loss value for the mixture network remains stable throughout the descent in contrast to the other two networks. The reconstruction results generated by the three models are depicted in Figure 10. It is clear that all three models achieve good reconstruction results, but the RLMN model produces the reconstructions of the quality that are closest in resemblance to the ground truth. Table 2 presents the quantitative results of the reconstructed images’ metrics of SSIM and PSNR. The information presented in the table reveals the RLMN model’s superior performance over the other two models in both intensity and phase image metrics, demonstrating the effectiveness of the RLMN model.

### 3.4. Comparative Experiment under Identical Noise Conditions

**Figure 11:**
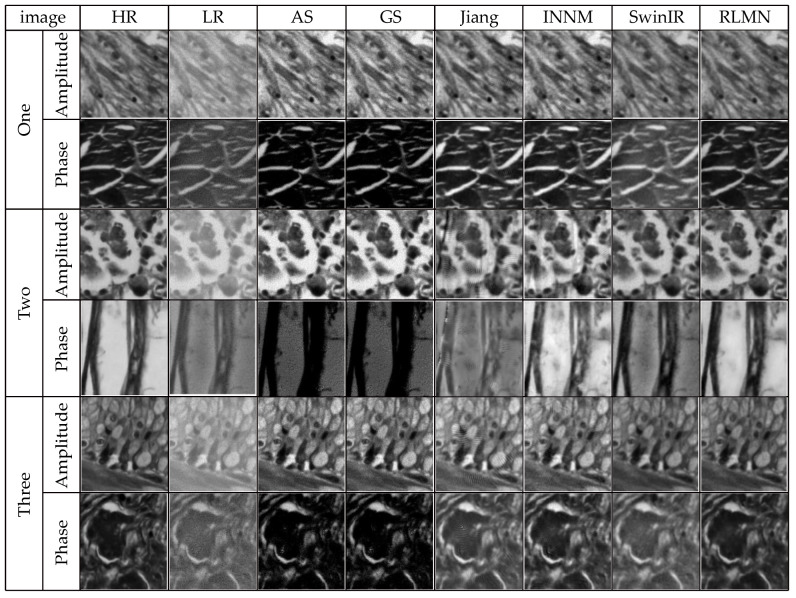
Reconstruction results under identical noise situations.

In the context of FPM image acquisition, the image quality can be compromised by a range of variables, like instrumentation and illumination, resulting in noise. To simulate these real-world conditions, this section employs Gaussian noise with a mean of 0 and a standard deviation of 3 × 10^−4^ as the primary interference condition to additionally validate the efficacy of the presented approach. The traditional phase recovery methods A-S [5] and G-S [5], the neural network-based reconstruction method proposed by Jiang et al. [8], the INNM method proposed by Zhang et al. [13], and the SwinIR method proposed by Wang et al. [12] were used as comparative algorithms against our proposed reconstruction method in this study. A trio of image sets are randomly picked from the test collection. The reconstruction outcomes of disparate approaches under identical noise conditions are illustrated in Figure 11. Table 3 presents the quantitative performance of the distinct reconstruction approaches using SSIM and PSNR evaluation. The results shown in Figure 11 and Table 3 indicate that the reconstruction approach presented in this article demonstrates a better reconstruction effect and performance metrics. The reconstructed images have higher clarity compared to the phase reconstruction results of the G-S, A-S, and Wang et al. methods. Relative to the methods of Jiang et al. and Zhang et al., the reconstruction results in this work do not exhibit significant artifacts, and they contain more textural details while reducing errors. The bolded values in Table 3 indicate the optimal results. In terms of the reconstruction metric values, the method in this chapter outperforms the other five approaches.

### 3.5. Comparative Experiment under Different Noise Conditions

**Figure 12:**
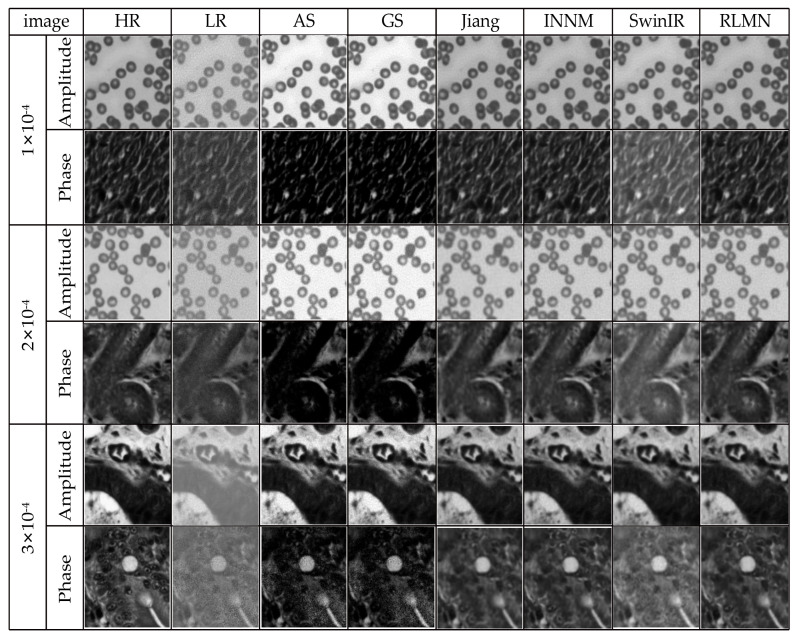
Reconstruction results comparison under dissimilar noise situations.

In real-world reconstruction scenarios, the noise levels can vary. Therefore, this section simulates the reconstruction outcomes and performance metrics of identical images under dissimilar noise situations. The noise magnitudes employed are 1 × 10^−4^, 2 × 10^−4^, and 3 × 10^−4^. A random selection of images is taken from each of the three test datasets. Figure 12 showcases the reconstruction outcomes of the identical amplitude and phase images under dissimilar noise situations, and Table 4 presents the quantitative reconstruction outcomes of the identical amplitude and phase images under dissimilar noise scenarios. The results presented in Figure 12 and Table 4 indicate that the proposed reconstruction method demonstrates superior results compared to the other five reconstruction methods, proving the effectiveness of this model.

### 3.6. Comparative Experiment on Real Images

**Figure 13:**
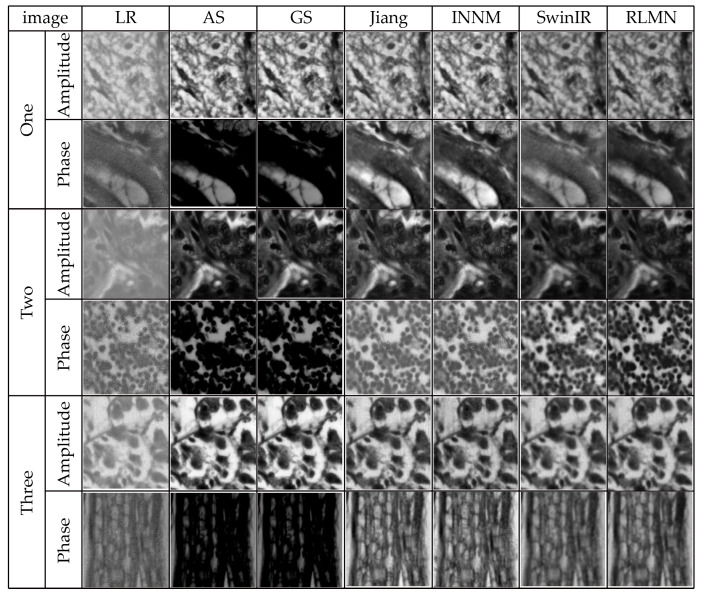
Reconstruction results of real data.

To validate the performance of the proposed network on real data, real picture data collected using the FPM system is combined with the simulated dataset to train the network. This section uses traditional algorithms A-S [5], G-S [5], and the techniques of Jiang et al. [8], Zhang et al. [13], and Wang et al. [12] as comparative experiments. The reconstruction results using actual data are illustrated in Figure 13. It is clear from the figure that the proposed method maintains better reconstruction performance and visual quality when tested on real data. Unlike the other five approaches, the approach presented in this work is able to effectively remove the coherent artifacts generated during the imaging process while also demonstrating higher clarity and more distinct textural details. Table 5 presents the time metrics for the reconstruction results of real data. As indicated by the table, the proposed approach requires the shortest reconstruction time compared to the other five approaches, with SwinIR being the second shortest. Although the A-S and G-S approaches have relatively short reconstruction times, their phase image quality is poor. In contrast, the approaches of Jiang et al. and Zhang et al. produce high-quality reconstruction images but require longer reconstruction times. These experimental results confirm the proposed approach’s superior performance.

## 4. Conclusions

In this article, constructed upon a residual local mixture network architecture, a reconstruction approach for Fourier ptychographic microscopy is put forward. In order to address the issues of poor reconstruction quality and artifacts in traditional reconstruction algorithms, the network incorporates a mixture attention mechanism and the Gaussian diffusion model. Continuous training of the network aims to achieve high-resolution image reconstruction. Through the experimental investigation, the effectiveness of the proposed approach is proven using datasets under identical noise, dissimilar noise, and real data. By comparing traditional algorithms and neural network-based methods, the proposed method’s efficacy is proven. Quantitative analysis of the reconstruction results is performed using SSIM, PSNR metrics, and time from different perspectives.

## Figures and Tables

**Table 1:** Quantitative results for different optimizers.

Image	Adagrad PSNR (dB) /SSIM	Adamax PSNR (dB) /SSIM	AdamW PSNR (dB) /SSIM
Amplitude	28.00 /0.6033	33.95 /0.9305	34.91 /0.9534
Phase	24.10 /0.5268	23.84 /0.8892	31.90 /0.9568

**Table 2:** Quantitative results for different models.

Image	RLDN PSNR (dB) /SSIM	RLMAN PSNR (dB) /SSIM	RLMN PSNR (dB) /SSIM
Amplitude	34.33 /0.9511	34.48 /0.9508	35.25 /0.9636
Phase	19.73 /0.9344	24.11 /0.9358	25.51 /0.9568

**Table 3:** Quantitative results of reconstruction under identical noise situations.

Image		AS PSNR (dB) /SSIM	GS PSNR (dB) /SSIM	Jiang PSNR (dB) /SSIM	INNM PSNR (dB) /SSIM	SwinIR PSNR (dB) /SSIM	RLMN PSNR (dB) /SSIM
One	Amplitude	19.39 /0.5218	19.40 /0.5245	25.72 /0.9123	23.73 /0.8732	33.81 /0.9203	35.47 /0.9615
	Phase	18.98 /0.3462	18.95 /0.3433	22.63 /0.9223	22.51 /0.8890	15.90 /0.6971	31.82 /0.9612
Two	Amplitude	18.09 /0.5336	18.09 /0.5379	25.34 /0.8540	23.17 /0.8239	29.75 /0.8839	35.25 /0.9636
	Phase	7.25 /0.1214	7.24 /0.1209	17.52 /0.7145	19.24 /0.7821	12.19 /0.7386	25.51 /0.9568
Three	Amplitude	20.15 /0.5318	19.90 /0.5336	21.87 /0.8278	19.89 /0.8553	34.64 /0.9246	35.91 /0.9630
	Phase	18.06 /0.3017	18.03 /0.2997	21.92 /0.8088	23.41 /0.8809	15.42 /0.6945	30.21 /0.9485

**Table 4:** Quantitative results of reconstruction under dissimilar noise situations.

Image		AS PSNR (dB) /SSIM	GS PSNR (dB) /SSIM	Jiang PSNR (dB) /SSIM	INNM PSNR (dB) /SSIM	SwinIR PSNR (dB) /SSIM	RLMN PSNR (dB) /SSIM
1 × 10^−4^	Amplitude	14.27 /0.5692	14.26 /0.5668	17.87 /0.7975	19.51 /0.7406	27.09 /0.8916	**39.80** **/0.9761**
	Phase	19.99 /0.3437	20.00 /0.3449	24.53 /0.9312	26.46 /0.9165	18.70 /0.5780	**29.58** **/0.9426**
2 × 10^−4^	Amplitude	16.95 /0.5155	16.97 /0.5157	21.71 /0.8686	19.77 /0.7516	33.54 /0.8979	**34.71** **/0.9467**
	Phase	15.98 /0.2410	15.97 /0.2384	20.03 /0.8930	21.63 /0.8931	17.27 /0.7961	**32.67** **/0.9642**
3 × 10^−4^	Amplitude	20.19 /0.6137	20.20 /0.6107	23.50 /0.8957	24.07 /0.8781	33.84 /0.9346	**35.80** **/0.9645**
	Phase	16.76 /0.2516	16.80 /0.2534	20.16 /0.8054	19.31 /0.7462	16.98 /0.7365	**26.46** **/0.9483**

**Table 5:** Time Metrics for real data reconstruction.

Model	Iterations	Reconstruction Time
AS	50	2.4987 s
GS	50	2.5133 s
Jiang	50	89.49 s
INNM	50	398.30 s
SwinIR	0	0.597 s
RLMN	0	0.315 s

## Data Availability

Data sharing is not applicable to this article.
